# Effects and Moderators of Computer-Based Training on Children's Executive Functions: A Systematic Review and Meta-Analysis

**DOI:** 10.3389/fpsyg.2020.580329

**Published:** 2020-11-26

**Authors:** Yifei Cao, Ting Huang, Jipeng Huang, Xiaochun Xie, Yuan Wang

**Affiliations:** School of Psychology, Northeast Normal University, Changchun, China

**Keywords:** computer-based training (CBT), executive functions (EFs), children, game-element, meta-analysis

## Abstract

Computer-based training has attracted increasing attention from researchers in recent years. Several studies have found that computer-based training resulted in improved executive functions (EFs) in adults. However, it remains controversial whether children can benefit from computer-based training and what moderator could influence the training effects. The focus of the present meta-analysis was to examine the effects of computer-based training on EFs in children: working memory, cognitive flexibility, and inhibitory control. A thorough search of published work yielded a sample of 36 studies with 216 effect sizes. The results indicated that computer-based training showed moderate training effects on improving EFs in children (*g* = 0.35, *k* = 36, *p* < 0.001), while training effects of working memory were significantly higher. Furthermore, we found near-transfer effects were marginally significantly higher than far-transfer effects. The standard training method was significantly more effective than training with game elements. In computer-based training, typically developing children had significantly better training effects than atypically developing children. Some additional factors, such as the number of training sessions and age, also modulated the training effects. In conclusion, the present study investigated the effects and moderators of computer-based training for children's EFs. The results provided evidence that computer-based training (especially standard training) may serve as an efficient way to improve EFs in children (especially typically developing individuals). We also discussed some directions for future computer-based training studies.

## Introduction

There is a consensus among researchers that executive functions (EFs) are the core skills critical for the cognitive, social, and psychological development of individuals (Lezak, 1982; Lyon and Krasnegor, 1996; Espy and Kaufman, 2002). In the present study, we adopted the three-factor model of basic EFs components: working memory, cognitive flexibility, and inhibition (Diamond, 2013), and higher-order EFs components, including reasoning, problem-solving, and planning. The model was built from the basic components (Collins and Koechlin, 2012; Lunt et al., 2012). The basic EF components can be explained in detail as follows: (1) working memory involves holding information in mind and mentally working with it (Diamond, 2013); (2) inhibition (also called inhibitory control) is the ability to neglect unrelated stimuli while concentrating on a specific stimulus, and suppress, stop, or delay behaviors according to the purpose (Karbach and Unger, 2014); (3) cognitive flexibility refers to the process of controlling the transformation between two tasks under the same cognitive resource, including the switching of attentional focus, cognitive tasks, and responses (Collette and Van der Linden, 2002; Diamond, 2006). Generally, EFs refer to individuals' psychological competence and the process by which they consciously monitor their own thoughts and behaviors (Zelazo and Müller, 2002; Li et al., 2004). Further, EFs are the abilities that make individuals responsible for making plans, continuously focusing attention and inhibiting distractions, memorizing and keeping information, flexibly shifting roles, and exercising self-control (Barkley, 2001; Blair, 2002; Richmond et al., 2011). Taking into consideration the significant role of EFs, researchers paid much attention to the possibility of improving EF skills.

In recent years, numerous studies have investigated the training and transfer effects on the EFs in adults and reached controversial conclusions on the efficacy of EF training (Kueider et al., 2012; Lampit et al., 2014; Melby-Lervag et al., 2016; Cao, 2018; Mayer et al., 2019; Sala et al., 2019b). However, comparatively, there are few studies on the training that focused on the EFs in children. Indeed, it is important to investigate the training efficacy on children's EFs, since great changes are happening during the growth of children's brain (Diamond and Lee, 2011). During childhood and adolescence, the behavioral and neural plasticity is particularly high, and the brain regions serving EF (i.e., the prefrontal lobe) are specifically sensitive to environmental influences (Bull et al., 2011). Furthermore, the findings on empirical studies and meta-analysis show converging evidence of stronger training and transfer effects on children than on adults (Karbach and Kray, 2009; Karbach and Unger, 2014; Zhao et al., 2016; Oberste et al., 2019). Therefore, we included children as the target age group to investigate the characteristics of EF training effect.

Various approaches such as mindfulness meditation (Zeidan et al., 2010; van de Weijer-Bergsma et al., 2012; Westbrook et al., 2013), aerobic exercise (Kamijo et al., 2009; Crush and Loprinzi, 2017; Wang et al., 2019), and computer-based training (Basak et al., 2008; Owen et al., 2010; Nouchi et al., 2013) have found significant training effects on the plasticity of EFs in adults. However, because of the different degrees of the prefrontal cortex brain maturation (Karbach and Unger, 2014) and the data on behavioral measurements (Karbach and Kray, 2009; Zhao et al., 2016), it is important to figure out whether these training approaches could be generalized and applied to the younger age groups. Several training approaches have been tested to investigate the training effects of EF in children. For example, mindfulness meditation training was found to be effective in young children (Flook et al., 2015; Li et al., 2019). Similarly, scholars reached a consensus that aerobic exercise (or exergame) has good transfer effects (Staiano et al., 2012; Chen et al., 2014) and could benefit the EFs in children. On the other hand, multiple studies have been conducted to examine the effects of computer-based training on the EFs in children; however, the empirical evidence has been mixed.

The first conflict in the present research is related to the transfer effects of computer-based training programs. EF impairments have been observed in children during neurodevelopmental disorders, including attention-deficit/hyperactive disorder (ADHD) and autism spectrum disorder (ASD). Therefore, EF impairments may place constraints on other cognitive functions, and this indicates that EF training could lead to transfer effects in other untrained executive processes and cognitive functions. In detail, near-transfer effects refer to the effects of cognitive interventions on various tasks tapping onto the same trained cognitive mechanisms, whereas far-transfer effects refer to the effects of training on various aspects of behavior and learning or different domain of EFs (Kassai et al., 2019; Scionti et al., 2020). For example, in the present meta-analysis, if the training is aimed at improving working memory, we define near-transfer effects to be the effects on working memory measurement, whereas far-transfer effects to be the effects on inhibition and flexibility measurement. Despite the nature of EFs, the transfer effects also depend on the characteristics of training. For example, training approaches such as aerobic exercise and mindfulness have various transfer effects because they involve many executive functioning processes. Because computer-based training is a type of explicit training (Takacs and Kassai, 2019)—that is, it has a specific training domain—it is less possible for it to have transfer effects on other cognitive functions, although there is no consensus regarding it. As an example, the results of the studies of Bigorra et al. (2016) and de Vries et al. (2015) suggested that computer-based training has no far-transfer effects but only near-transfer effects. On the other hand, previous research also indicated that the effects of computer-based training could transfer to an untrained domain (Goldin et al., 2014; Liu et al., 2015). In fact, most cognitive training programs are designed to improve not only the specified domain but also the general cognitive ability or, at least, some core cognitive mechanisms (Sala et al., 2019a). Therefore, it is vital to examine whether computer-based training is effective for far-transfer effects.

The reason for the difference in the results may be because of the difference in the plasticity of the three aspects of EFs. In particular, several empirical studies have investigated the training effects of computer-based training on children's working memory; some studies found the computer-based training to be effective (Prins et al., 2011; Dunning et al., 2013; Rojas-Barahona et al., 2015; see review by Klingberg, 2010; Morrison and Chein, 2011; Spencer-Smith and Klingberg, 2015), whereas some studies also found the training effect to be insignificant (Wong et al., 2014; de Vries et al., 2015; Melby-Lervag et al., 2016). There were also contradictory results about the transfer effects on inhibition; some studies indicated that computer-based training cannot be transferred to inhibition (Spierer et al., 2013; Ackermann et al., 2018; Hessl et al., 2019), whereas others found the transfer effects to be significant (Blakey and Carroll, 2015; Sanchez-Perez et al., 2018). For flexibility, only one study showed significant transfer effects (Espinet et al., 2013), and most found insignificant results (Egeland et al., 2013; de Vries et al., 2015; Weerdmeester et al., 2016).

Another disagreement addressed in computer-based training is whether training, including game elements, could enhance the training effects (Doerrenbaecher et al., 2014; Johann and Karbach, 2019). Traditional training refers to training programs using standard cognitive tasks (e.g., Corsi block-tapping task, N-back task) to enhance individuals' cognitive ability (e.g., Espinet et al., 2013; Zhang et al., 2019). Game-based training (e.g., Cogmed, Braingame Brain) differentiates from traditional training by using multiple sensory modalities (color, sounds, movement), providing immediate feedback (quality and accuracy), and includes animated characters, narratives, interactive environments, and player advancement through different levels to make standard cognitive tasks more interesting (Prins et al., 2013). According to self-determination theory (Ryan and Deci, 2000), adding game elements to the training environment could induce children's intrinsic interest in the training task. In comparison to the standard training, game-based training provides trainees with more timely feedback and more interesting training content and storyline; it also better stimulates the motivation of individuals during the training (Wang et al., 2019). Several studies have utilized computer-based training to investigate the training and transfer effects on adults. According to Wang et al. (2019), computer-based training that includes game elements could significantly enhance working memory and cognitive flexibility in adults, but not inhibition; however, there is also a review study that reached a conclusion that the effects of game-based training such as Cogmed on individuals' working memory are unsubstantial (Shipstead et al., 2012). Recently, there has been an increasing interest in using game elements in EF training for children (Klingberg et al., 2005; Alloway et al., 2013; Dunning et al., 2013; de Vries et al., 2015; Homer et al., 2017). However, the number of studies is fewer for children than for adult training research, and there is a lack of review and meta-analysis research. Thus, there is a mixed view about whether the inclusion of game elements could enhance training effects on EFs in children. However, some studies found that using game elements could enhance intrinsic motivation during the training process (Johann and Karbach, 2019), but the results are mixed about this as well.

In addition to the variables of the training program, participants' clinical risk status can also influence the training effects, and there is a controversy between the two accounts regarding this issue. The magnification account proposes that individuals who are already performing well will also benefit more from cognitive interventions, and this indicates that typically developing children might benefit more than atypically developing ones (Björklund and Douglas, 1997; Brehmer et al., 2007). On the other hand, the compensation account assumes that high-performing individuals could benefit less from cognitive training, because they have less room for improvement, and this indicates that atypically developing children could benefit more from cognitive training (Jaeggi et al., 2008; Dahlin, 2011; Karbach and Verhaeghen, 2014). For example, children with ADHD and ASD may experience different benefits from training. We appreciate that difference exists between typically developing children and atypically developing children (children with developmental delay or functional disabilities). Many researchers believe that atypically developing children could gain more from EF training than that typically developing children would gain (Melby-Lervag et al., 2016). However, in the case of a standard training approach, things could be different. According to Diamond and Ling (2016), compared to other non-computerized training, standard training shows fewer benefits for EFs, and the potential reason for it might be the absence of in-person interaction. Moreover, operating computers using a keyboard and mouse might be a complicated process for children with low comprehension skills to understand, and this could possibly influence the training effects. On the basis of the meta-analysis of Takacs and Kassai (2019), computer-based training was significantly more beneficial to typically developing children than to atypically developing children. Therefore, to figure out the controversial conclusion and come to a consensus, systematic investigation is needed.

According to previous research, there are some other moderators that may affect the efficacy of computer-based training, and we included these moderators in the following meta-analysis. Initially, the training session and the duration were correlated with training and transfer effects of EFs (Primack et al., 2012; von Bastian and Oberauer, 2014; Schwaighofer et al., 2015). According to Bavelier et al. (2018), in the novice stage, the processing resources are used extensively in order to strengthen the individual's attention control and cognitive flexibility; therefore, the transfer effect enhances and expands to a wider range of learning strategies with the increasing training time, and the person benefits extensively in terms of cognitive improvement in the game, which is consistent with the learning to learn hypothesis (Bavelier et al., 2012). Finally, the training skills begin to enter the automatic state after a certain training duration is reached. At this stage, the training effect is still improving, but the attention control and cognitive flexibility required for learning are slowly diminishing, and hence the degree of transfer effect is decreasing.

The sample male percentage has also been considered as a moderator in computer-based training. Males and females have been noted to have different attitudes toward computer technology; furthermore, girls are less positive and less likely to enjoy playing different kinds of computer games than boys (Lucas and Sherry, 2004; Hartmann and Klimmt, 2006; Walkerdine, 2007; Homer et al., 2012; Powers et al., 2013). Therefore, boys might likely get benefit more from computer-based training because of their increased intrinsic motivation for computer technology. By investigating the different training effects on boys and girls, we can analyze the gender effects related to computer-based training; in addition, the influence of motivation on training effects can also be studied because there are differences in the motivation level and the attitude toward computers between boys and girls.

Mean sample age is the last moderator considered here. We selected the age group of the study participants as 3 to 12 years, which is the period when the neural system and the brain experience major development, and hence the plasticity of the brain and nervous system will differ in the different time periods (Nelson, 2000; Andersen, 2003). The participants aged from 0 to 3 years were not included because few computer-based training experiments have been done on young children of that age range. According to Karbach and Unger (2014), the latent factor structure of EFs changes qualitatively across the development period, from a unitary structure in preschoolers to multiple subcomponents in school-aged children and adolescents. Therefore, it is important to figure out the time period that is more effective to provide computer-based training to develop children's EFs.

In conclusion, the effect of computer-based training on children's overall EFs and the three aspects related to it need to be analyzed. Also, factors, including transfer effect, participants' clinical risk status, training type, number of training sessions, the training duration, mean sample age, and sample male percentage, may play moderating roles. Although previous studies have been done on relevant topics (Rapport et al., 2013; Robinson et al., 2014; Diamond and Ling, 2016), the present study would provide new knowledge by focusing on the effects and moderators of computer-based training and investigating the training and transfer effects on the three aspects of EFs separately. Furthermore, this study discusses the effect of game element as a moderator in computer-based training for the first time in a meta-analysis. Therefore, in the present study, we used meta-analysis to investigate the effects of computer-based training on EFs in children and the effects of different moderators. The purposes of the present meta-analysis are as follows:

- To examine the effects of computer-based training on EFs in children by synthesizing the overall effect sizes; and
- To examine the role of different moderators on the effect sizes of computer-based training on EFs in children.

## Methods

### Operational Definitions

We selected the EFs interventions based on computer, smartphone, tablet computer, and other electronic devices. Computer-based training is defined as an intervention that uses a computer to carry out conventional EFs tasks such as Corsi block-tapping (for working memory), dimensional change card sort (DCCS; for flexibility), and Stroop (for inhibition) tasks, and commercially accessible electronic programs with game elements such as Jungle Memory™, CogMed RM, and Braingame Brian (Prins et al., 2013), which all aimed at improving EFs of children.

For the outcome measures of EFs, we followed the approach of Takacs and Kassai (2019) to categorize them based on the main executive process used. Working memory measurements include content domains such as word, digit, spatial span-like tests, N-back tasks, and other tasks needed for the active manipulation of information stored in mind. Tasks that require rule switching are considered as flexible such as DCCS and gender-emotion switch tasks. We categorized go/no-go, flanker, and Stroop-like tasks as inhibition measurement tests because participants need to inhibit the distraction, pre-potent, and automatized response when performing the tasks (Scionti et al., 2020).

The study also aimed to explore the transfer effects of computer-based training; therefore, we labeled the transfer effect of each effect size as near transfer or far transfer, based on the study by Scionti et al. (2020). The effect sizes that measured the same aspect of EFs as interventions were defined as near-transfer, whereas those that measured the different aspects of EFs with the intervention were defined as far transfer.

### Search Strategy

**Figure 1:**
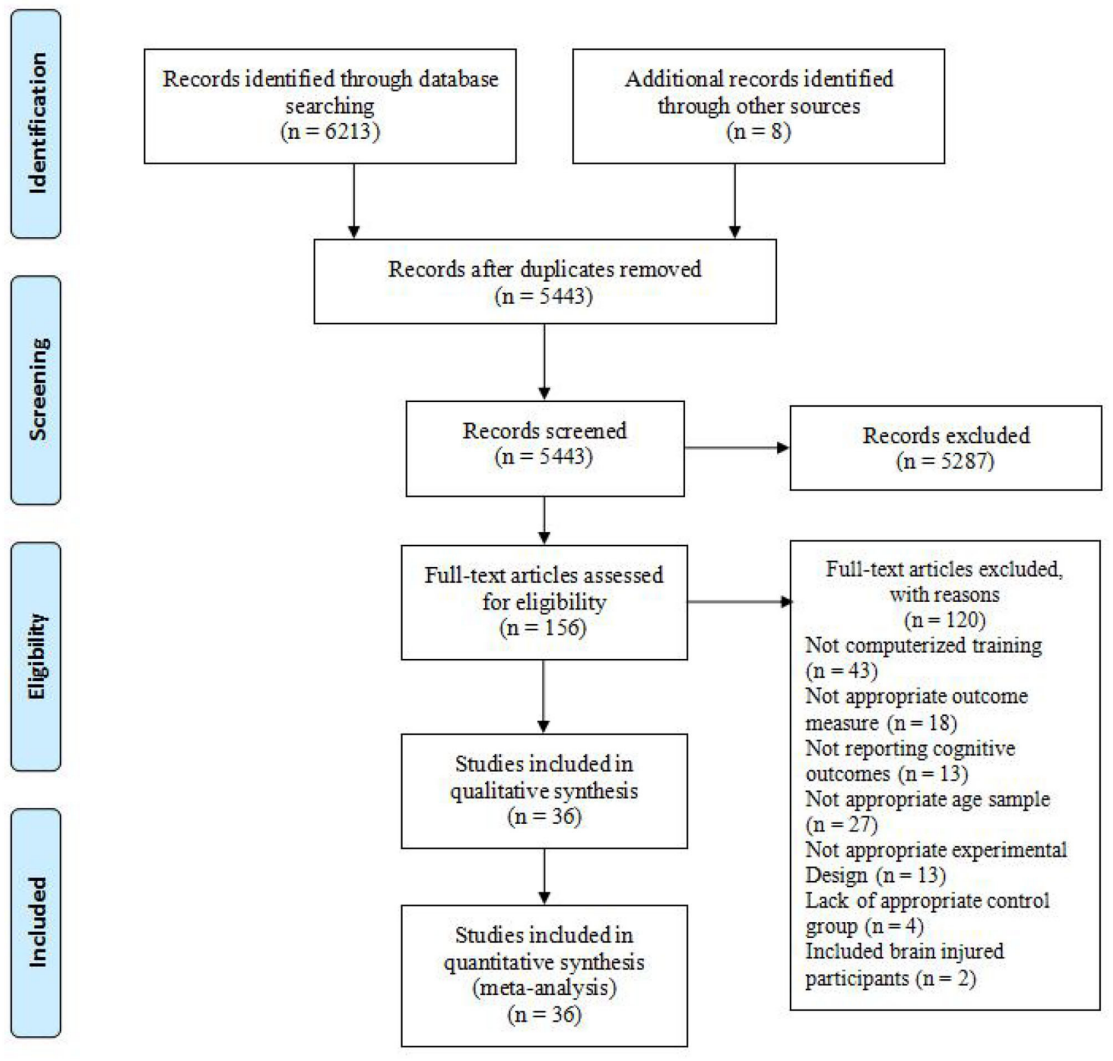
PRISMA flowchart for the include studies in the meta-analysis.

In accordance with the Preferred Reporting Items for Systematic Reviews and Meta-Analyses (PRISMA) statement (Moher et al., 2009), we used a systematic search strategy to find pertinent studies. And in order to include all the available sources that assessed the effects of computer-based training on the EFs of children, we searched several databases for published studies, including PsycINFO, PsycARTICLES, Scopus, Google Scholar, Social Sciences Citation Index, Web of Science, and Dissertations Online. We searched the entire text of English-written journal articles by using different combinations of the terms “executive functions” (e.g., “cognitive control” OR “behavioral control” OR “self-control” OR “effortful control” OR “self-regulat^*^” OR regulat^*^ OR “executive functi^*^” OR attention OR “working memory” OR inhibit^*^ OR planning OR “cognitive flexibility”), “computer-based training,” (e.g., “computer^*^ training” OR “computer game^*^ training OR “video game^*^ training” OR “video game^*^” OR “videogame^*^ training”), and “children” (e.g., “preschoolers” OR “preschool” OR “early childhood” OR “kindergartner” OR “teenager^*^” OR “youth^*^” OR “adolescen^*^”) and their synonyms from year 1950 to April 15, 2020. Second, we sent out personalized emails to the prominent servant to obtain unpublished manuscripts (Figure 1).

### Inclusion Criteria

The inclusion criteria for the study were as follows: (1) used computerized task or video game for EF training; (2) measured at least one EF outcome; (3) trained participants aged from 3 to 12 years; and (4) used pretreatment–posttreatment designs and randomized controlled trials with at least a control group (active or passive).

### Exclusion Criteria

The exclusion criteria were as follows: (i) the participants reported brain damage, or their mean age was not from 3 to 12 years; (ii) the training group did not earlier receive any computerized or video-game training; (iii) there was no control group; (iv) the study did not report a neurocognitive test of EFs; and (v) the study did not present enough data to calculate effect sizes.

### Coding

We documented the following information for identification and quality assessment of the study and for the statistical analyses afterward: bibliographic information (author's name, published year, country), characteristics of the sample (mean age, clinical risk status, with the reason for atypical categorization), sample size (number of participants in intervention group and control group), effect sizes between relevant indicators, characteristics of intervention (type of intervention, number of sessions, and training minutes), type of kid outcome (e.g., working memory, cognitive flexibility, and inhibitory control), transfer effect (e.g., near transfer and far transfer), and type of measurement (e.g., digit span, Stroop, flanker).

It should, however, be noted that some studies did not report the exact training period, so we decided to use the minimum time of the training for those studies for an estimation (i.e., Dunning et al., 2013; Bigorra et al., 2016). Furthermore, some studies reported more than one training condition that meet our criteria, and we included those studies for more contrasts. Following this process, we were able to achieve 100% consensus rate for every single piece of data extracted. The second and third authors of this study each separately coded all the studies included in this meta-analysis. The initial interrater reliability, calculated across all primary studies, was almost 1.0. The discrepancies, if any, were identified and resolved before the statistical analyses.

### Meta-Analytic Procedures

Comprehensive Meta-Analysis (CMA) software, version 3.0 (Borenstein et al., 2005), was used to calculate the effect sizes in each study. We selected Hedge's *g* instead of Cohen's *d* because the former corrects for small samples (Borenstein et al., 2009). Effect sizes were calculated using means and SDs of the posttest (or follow-up test) for both the training group and control group; *p*-values, *t-*values, *f*-values, and other statistics that reflect the difference were used when means and SDs were unavailable. Effect sizes were combined using CMA software when a study reported more than one appropriate outcome measures. The present meta-analysis aggregated the effect sizes of the posttest and follow-up tests. The random-effects model was selected to compute the average effect sizes as the outcomes included different kinds of measurement.

In order to investigate the possibility of publication bias, we (a) examined funnel plots and *p* curve plot, (b) calculated Rosenthal's fail-safe N to find how many null findings would be needed to turn the average effect sizes into insignificant, (c) examined Egger's test, and (d) conducted trim-and-fill analysis when the funnel plot was asymmetrical.

We analyzed several potential moderators in the meta-analysis. Subgroup analysis was used for categorized moderators, including training type (standard training and game-based training), transfer effect (near transfer and far transfer), and development at risk (typical development and atypical development). We conducted metaregression analysis for continuous moderators, including the number of training sessions, total training time (minutes), mean sample age, and sample male percentage.

## Results

### Results of Primary Meta-Analysis on Posttest

#### Selected Studies

The final search results contained 36 studies (including 216 effect sizes) in the qualitative and quantitative synthesis of this review (see detailed selection process in the form of a PRISMA diagram in Figure 1). There were 2,585 participants included in the present meta-analysis. The characteristics, outcome measure, transfer effects, posttest effect sizes, and follow-up test effect sizes are presented in Table S1 (see in Supporting Information section).

In summary, 18 studies in the present meta-analysis used traditional computer-based training, and the other 18 studies used computer-based training with game elements. In the traditional computer-based training studies, researchers used different kinds of cognitive tasks to improve children's EFs (e.g., DCCS and N-back task). While in the game-based training, game elements were added into the cognitive tasks to make computer-based training more interesting (e.g., Cogmed and Braingame Brain). For the control conditions, 10 studies used passive control, and participants did not receive any intervention; 23 studies used active control, and participants received non-adaptive or unrelated intervention; three studies used both passive and active control. And for the measurement tasks used in the included studies, we made a summary table (Table 1).

**Table 1:** The summary of outcome measurements used in the included studies.

**Component of EFs**	**Outcome measurement**	**Times**
Working memory	Digit span task	17
	Corsi block-tapping task	6
	Spatial span task	5
	Leiter-revised spatial working memory task	4
	Word span task	4
	Counting span task	4
	Span board task from WAIS-R-NI	3
	Odd One Out from the Automated Working Memory Assessment (AWMA)	2
	Mr.X from AWMA	2
	Letter–number sequencing task	2
	Listening recall task	2
	Dot matrix	1
	Block recall task	1
	Processing letter recall	1
	Shape recall task	1
	N-back task	1
	Navigation span task	1
	Working memory span backwards subtest of the WISC	1
	Sentence span task	1
Inhibition	Stroop	9
	Go/no-go	6
	Continuous performance test	6
	Stop-signal task	3
	Child attention network test	2
	Simon says	1
	Flanker task	1
	Delay of gratification	1
	Iowa gambling task	1
	Movement assessment battery for children	1
	Peg tapping task	1
Flexibility	Switching task	5
	Trail making tests	4
	Dimensional change card sort	3
	Tower of London	2
	Wisconsin card sorting test-64 (WCST-64)	1
	The heart–flower Stroop task	1
	Intra–extra dimensional set shift	1
	Flexible item selection test	1
	Flexibility false alarms	1
	Dots task	1

For the clinical risk status of children, 18 studies included typically developing children, and the other 18 studies included atypically developing children. Among the studies included atypically developing children, 12 studies included children diagnosed with ADHD, two studies included children with low working memory, one study included children with learning difficulty, one study included children diagnosed with fragile X syndrome, and one study included children with developmental dyslexia.

#### The Overall Training Effects

**Figure 2:**
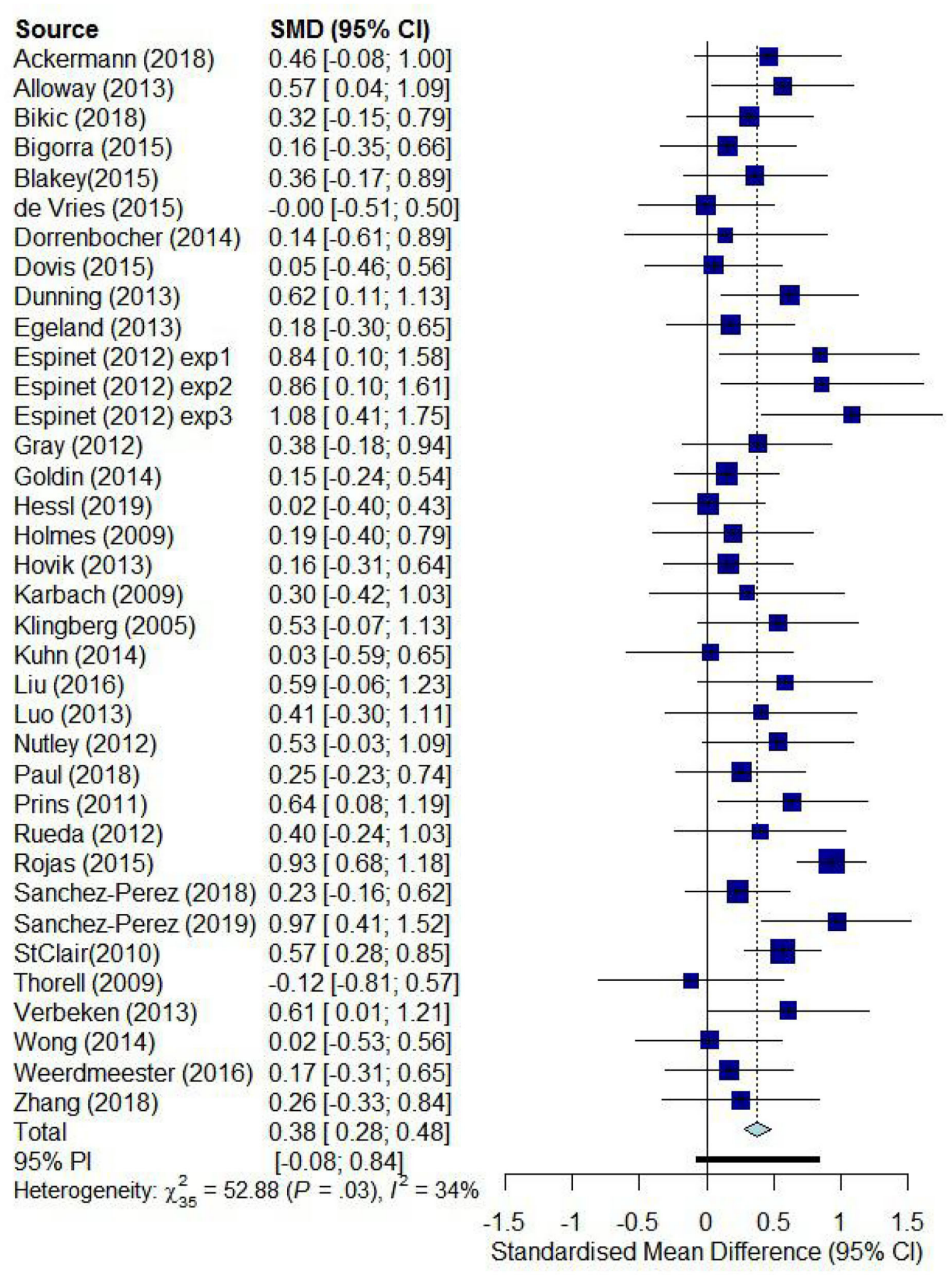
Overall efficacy of computer-based training on children's EFs.

All the 36 studies reported posttest results, and we aggregated the effect sizes from different studies. Random-effects model showed that the effect of computer-based training on EFs in children was 0.36, 95% confidence interval (CI) = [0.26, 0.47], *p* < 0.001, *Q* = 64.31, *p* < 0.001, *I*^2^ = 47.13, tau = 0.21. In addition, 12 studies reported follow-up test results; we aggregated the different effect sizes. Random-effects model showed that the effect of computer-based training on EFs in children's follow-up test was 0.22, 95% CI = [0.08, 0.36], *p* < 0.01, *Q* = 12.20, *p* = 0.35, *I*^2^ = 9.87, tau = 0.08. Then, we compared the effect sizes of posttest and follow-up test and found no differences; *Q* = 2.55, *df* = 1, *p* = 0.11. Next, we aggregated the posttest and follow-up test to examine the total effect of computer-based training on EFs in children; the results indicated that the random effect was 0.35, 95% CI = [0.25, 0.45], *p* < 0.001, *Q* = 75.32, *p* < 0.001, *I*^2^ = 53.53, tau = 0.21 (Figure 2). For the total effect, we conducted several meta-analyses of different aspects of EFs and moderator effects.

### Other Meta-Analyses for Different Aspects of EFs

In the next step, we further conducted a specific analysis to examine the effects of computer-based training, taking into consideration the moderators, because the present meta-analysis contained three aspects of EFs: working memory, flexibility, and inhibition (Table 2). There was a significant difference between the effect sizes of the three EFs aspects; *Q* = 16.82, *df* = 2, *p* < 0.001. Further analysis revealed that training effect on working memory was significantly higher than flexibility, *Q* = 16.46, *df* = 1, *p* < 0.001; and inhibition, *Q* = 5.77, *df* = 1, *p* < 0.05.

**Table 2:** The effect sizes for different aspects of EFs.

	***k***	**Hedge's *g***	**95% CI**	**tau**	***I*^**2**^**	***Q***
Working memory	29	0.41	[0.28, 0.54]	0.26	5681%	62.51^***^
Flexibility	14	0.13	[−0.04, 0.29]	0.21	46.06%	24.10^*^
Inhibition	22	0.25	[0.14, 0.35]	0.12	21.87%	26.88

* p < 0.05,

*** *p < 0.001, k = the number of the effect sizes*.

### Potential Moderators in Meta-Analysis

The purpose of the study was to investigate the role of several potential moderators on the training effects. The potential moderators included training type, transfer effect, clinical risk status, control condition, number of training sessions, total training time, mean age, and the number of males in the training group. Test of moderators was reported for categorical moderators, and metaregression was used for continuous moderators (Table 3).

**Table 3:** Analysis of potential moderators of effect sizes in the posttest.

**Moderator variables**	***k***	**Hedge's *g***	**95% CI**	***Q***
Training type				
Standard training	18	0.46	[0.30, 0.62]	4.96^*^
Game-based training	18	0.24	[0.14, 0.35]	
Clinical risk status				
Typically developing	18	0.47	[0.30, 0.63]	5.64^*^
Atypically developing	18	0.23	[0.13, 0.33]	
Transfer effect				
Near transfer	34	0.27	[0.20, 0.34]	2.93
Far transfer	16	0.18	[0.10, 0.26]	
No. of sessions	37	−0.02	[−0.03, 0.00]	6.30^*^
No. of minutes	29	0.00	[0.00, 0.00]	3.12
Sample male percentage	35	−0.75	[−1.74, 0.24]	2.21
Mean sample age	37	−0.05	[−0.09, −0.01]	5.79^**^

* p < 0.05,

** *p < 0.01, k = the number of the effect sizes*.

The results for the categorical moderators showed that mean effect sizes for the standard training group [Hedge's *g* = 0.46, 95% CI = (0.30, 0.62)] were significantly higher than those in the game-based training group [Hedge's *g* = 0.24, 95% CI = (0.14, 0.35)], *Q* = 4.96, *df* = 1, *p* < 0.05. Typically developing children gained significantly more training effects [Hedge's *g* = 0.47, 95% CI = (0.30, 0.63)] than atypically developing children [Hedge's *g* = 0.23, 95% CI = (0.13, 0.33)], *Q* = 5.64, *df* = 1, *p* < 0.05. The mean effect sizes of near transfer [Hedge's *g* = 0.27, 95% CI = (0.20, 0.34)] were marginally significantly higher than far transfer [Hedge's *g* = 0.18, 95% CI = (0.10, 0.26)], *Q* = 2.93, *df* = 1, *p* = 0.09.

The metaregression analysis showed that the number of sessions (slope = −0.02, *Q* = 5.01, *p* < 0.05) and mean sample age (slope = −0.05, *Q* = 6.95, *p* < 0.01) were significantly negatively correlated with effect sizes, whereas number of minutes (slope = 0.00, *Q* = 3.34, *p* = 0.10) and sample male percentage (slope = −0.51, *Q* = 1.31, *p* = 0.25) showed no effect.

### Publication Bias Testing

**Figure 3:**
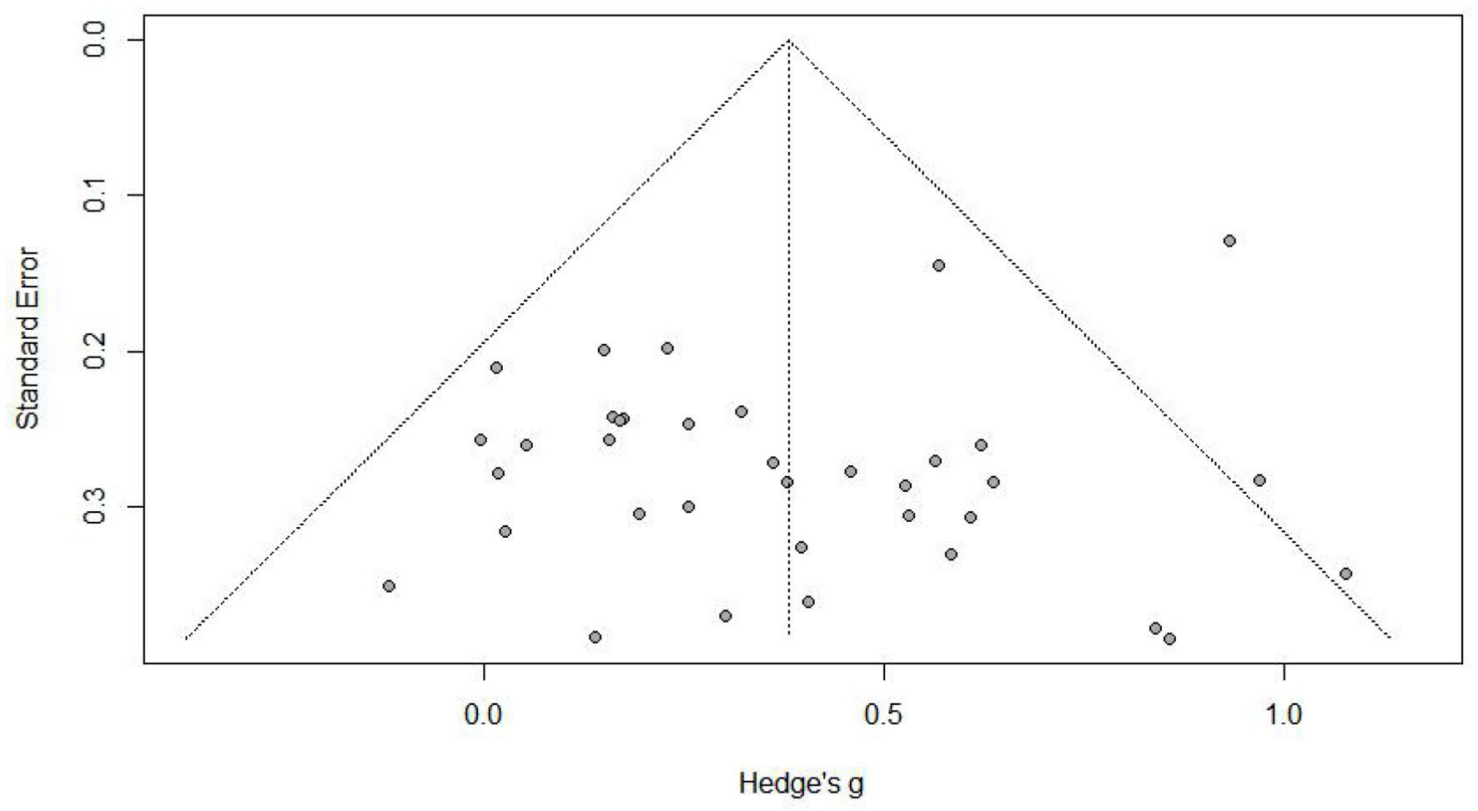
Funnel plot for the posttest results.

In the study, we first used the funnel plot to examine the publication bias. The funnel plot (Figure 3) shows no indication of publication bias, as the effect sizes are shaped roughly like a funnel, and only a few studies fall outside of the triangular region of the pseudo–confidence interval. Next, for the purpose of testing the publication bias more precisely, we conducted Rosenthal's fail-safe N and Egger's test.

The assessments of Rosenthal's *N*-test confirmed that there was no publication bias (fail-safe *N* = 904); the number of N demonstrates that extra 904 insignificant studies would be needed in order to invalidate the findings. Egger's test corroborated this appraisal (*p* = 0.11).

**Figure 4:**
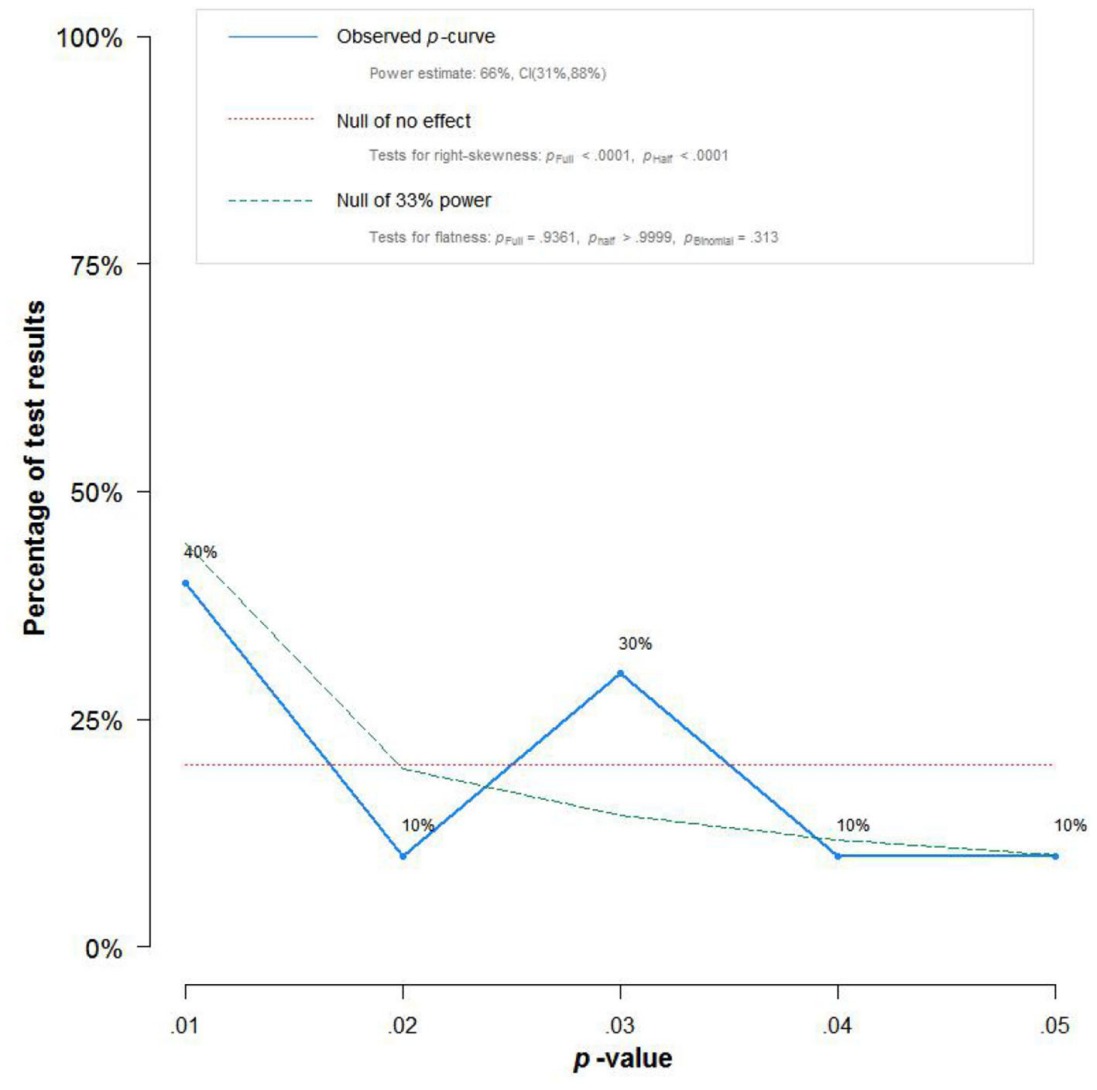
*p* curves: the solid line is the distribution of *p*-values in the study. The dotted line shows the expected distribution of *p*-values if there was no effect. The horizontal line shows the expected *p* curve under 33% statistical power.

Trim-and-fill (Duval and Tweedie, 2000) method was used to examine the effects of publication bias on the results. Based on the results, the training effect was still significant (*p* < 0.001) after adjustment using random-effects model. We also examined the *p* curve, and according to Simonsohn et al. (2014), the right-skewed *p* curve represents a robust and true effect size. The right-skewed *p* curve plot (Figure 4) indicates that there is a strong relationship between computer-based training and EFs in children.

## Discussion

The present study conducted a meta-analysis with the aim to investigate the training and transfer effects and moderators of computer-based training on EFs in children. Our results indicated that computer-based training has a moderate effect size on EFs in children, and the transfer effect is marginally more pronounced in near-transfer conditions. Typically developing children gained more improvement during training, but adding game element could decrease the training and transfer effects. The number of sessions and sample age also moderated this process.

First, the results suggested that computer-based training has a moderate to small effect on EFs in children; posttest and follow-up test effects were equal. The finding of this study is consistent with those reported in the previous meta-analysis on the effect of computer-based training on EFs in children (Melby-Lervag and Hulme, 2013; Melby-Lervag et al., 2016; Takacs and Kassai, 2019). Despite various new training approaches emerging such as school curriculum (Blair and Raver, 2014; Dias and Seabra, 2015), art activities (Thibodeau et al., 2016), and strategy learning (Deano et al., 2015; Nash et al., 2015), and, as Diamond and Ling (2019) pointed, despite computer-based training showing low efficiency than other training approaches because of the absence of in-person interaction between the trainer and the trainee, computer-based training has proven to be effective in training EFs in children. Furthermore, the magnification effect of computer-based training in the present study was inconsistent with the result in Diamond and Ling (2019). The reason could be the different age range of selected participants, because the age range in the present study is 3 to 12 years, whereas Diamond and Ling also included adults. According to the results of the present study and some previous studies, younger children could benefit more from cognitive training than adults because of the high plasticity of the brain and more room for cognitive improvement (Cepeda et al., 2001; Kray et al., 2008; Karbach and Kray, 2009). The lower training effects of adult participants may have decreased the effect size of the whole sample; therefore, a difference between the effect sizes of the present study and Diamond and Ling (2019) was observed. Most of the computer-based training can be considered as explicit training, aiming at improving EFs specifically, which is different from other training approaches that do not use EFs directly; therefore, computer-based training is still an effective training approach for EFs.

Second, our findings showed that in computer-based training, there is a significant difference in training and transfer effects on the three aspects of EFs in children. The effects on working memory and cognitive flexibility were significant; however, on inhibition, they were non-significant. These findings are consistent with the results of a recent review of the adult group (Wang et al., 2019). The common demand theory explains the different training and transfer effects on the three aspects (Dahlin et al., 2008). The theory emphasized that computer-based training could transfer to the EF domains with the same cognitive demands of the training tasks, but not to those without the same demands. As a result, the insignificant training and transfer effect on children's cognitive flexibility is likely because most computer-based training tasks were not designed to include children's inhibition during training. Besides this, the results of the present meta-analysis also indicated that the training and transfer effects on working memory were significantly higher than those on inhibition and flexibility. The possible explanation for this issue could be that more than two-thirds of the included studies were specifically aimed at improving children's working memory, while inhibition and flexibility were also tested as the examination of the far-transfer effects. It has been tested in the previous meta-analysis that near-transfer effects are more robust than far-transfer effect (Kassai et al., 2019), while at the same time, inhibition and flexibility in the present meta-analysis were more tested as far-transfer effects. As a result, the effect sizes of inhibition and flexibility were significantly lower than working memory.

In contrast to our hypothesis, the addition of game element in computer-based training undermined the training and transfer effects on children's EFs. As we mentioned earlier, researchers support that game element could enhance children's motivation on EF training; however, the present meta-analysis found that the training effects in the traditional standard training group were significantly more than in the game-element group. According to Doerrenbaecher et al. (2014), game element in computer-based training has dissociative effects on motivation and training effect. Therefore, increased motivation does not mean that the training effect could be better in the video-game group. The reason for decreased training effects in the game-based group could be that computer-based training itself is a well-designed approach for EF training, and so the addition of game elements might damage the training effects. It is also noteworthy that computer-based training with game elements is not the same as video games. According to Johann and Karbach (2018), training with game elements is adding storyline and games in the traditional training approaches. The addition of game element in the training program did not mean that it was now a video game, and it may cause children to feel tiresome during training. Therefore, the idea of gamification does not mean adding game elements to the standard training program, but instead means adding a training element in real video-game playing. For instance, Liu et al. (2015) used Fruit Ninja, a commercial video game, to train children's inhibition, and Diarra et al. (2019) used Super Mario to increase participants' oculomotor inhibition.

Moreover, we found that computer-based training had both near-transfer and far-transfer effects, and the near-transfer effect is marginally significantly better than the far-transfer effect. Although the far-transfer effect was significant, the effect size was small, which is in line with previous research (Kassai et al., 2019). Researchers, nowadays, tend to believe that cognitive training does not enhance the general cognition but only the specific aspect of cognitive function (Sala and Gobet, 2019). But in our research, training on one EF aspect could have a small transfer effect on the other EF aspects. This transfer effect could be explained by a meta-analysis of functional neuroimaging data, indicating that aspects of EFs partially overlap other neural networks (McKenna et al., 2017). Therefore, the training specified for one EF aspect could also improve other EF aspects because it overall improved the shared brain area.

Regarding the participant type, the findings indicate that typically developing children benefit more from computer-based training. However, previous empirical research and meta-analysis found that the performance of atypically developing children improved more than typically developing children (Peng and Miller, 2016; Scionti et al., 2020). Regarding the compensation theory, typically developing children benefit less because they are already functioning at the optimal level and therefore have less room for improvement. This is in line with the magnification account mentioned in the review of Karbach and Unger (2014). The magnification account is one of the two prominent accounts used in describing and explaining the individual differences in training-related performance gains, which assumes that individuals with high baseline performance will benefit most from cognitive interventions. In computer-based training, typically developing children are those with higher baseline performance, and they have more efficient cognitive resources to acquire and implement new strategies and abilities; therefore, they benefited more from training compared to atypically developing children (e.g., ADHD, ASD). Additionally, the elements of computer-based training might also be an explanation. Computers are used during training, and children need to operate it using the mouse and the keyboard, which could be complicated for atypically developing children to comprehend.

The results of the mean sample age suggest that younger children could benefit more from computer-based training. According to Wass et al. (2012), there is a negative correlation between age and transfer effects of cognitive training. This kind of negative correlation may be the result of the increasingly complex neural networks as children grow older, and thus training undifferentiated networks (among younger children) is more effective than training those already specialized (among older children). This is consistent with the results of the meta-analysis of Peng and Miller (2016), which suggested that younger participants showed greater benefits of attention training.

Consistent with the study by Powers et al. (2013), our findings indicate that the effect of sample male percentage as a moderator is insignificant. Motivation and training effects were dissociable and independent from each other (Doerrenbaecher et al., 2014); this means that a higher level of motivation does not guarantee better training effects. As a result, although boys were shown to be more interested in operating computers, their training-related improvement did not show a significant difference from that of girls. Combined with the results of game-based training, it is interesting to note that increased motivation for training did not improve the training and transfer effects, and therefore, motivation may not directly correlate with the training effects.

The present study also indicated that training effects decrease with the increase in the number of training sessions. Further, this negative effect of the number of sessions could be explained by the model raised by Bavelier et al. (2018), which is based on the inverted U-shape curve for generalization as training proceeds raised. As participants move through the early-to-intermediate phase of training, the task is expected to be demanding in terms of processing resources, resulting in enhancements in EFs, and as a result, greater training and transfer effects are expected. While, after some point, task functions begin to be automatized, although the performance of the training task itself increases with training, the transfer effects of training are less expected because automatization entails releasing demands on other EFs components. Therefore, we observed the decreased effect sizes with the increasing training sessions.

## Limitations

The current study is characterized by a number of limitations, suggesting avenues for future research. First, there were too few effect sizes included in the meta-analysis for research on flexibility, so we were unable to reach a convincing conclusion about the training and transfer effects on children's flexibility. The paucity of effect sizes caused publication bias and heterogeneity in the study. In future work, investigating the training effects on flexibility might prove useful. Second, the present meta-analysis did not contain unpublished studies because we did not find any, and so future studies should include unpublished results. Third, there were some studies that conducted several training sessions, and some of the effect sizes might potentially misestimate the training effects. A negative relationship between the number of training sessions and training effects was found, and it is hard to distinguish if the relationship was caused by the studies with few sessions.

In addition, in the present meta-analysis, we found there were more working memory training studies than inhibition training and flexibility training studies, and this may cause ambiguity in investigating the exact effect of computer-based training on the three aspects of EFs. One way to solve this issue is to conduct multivariate analysis and to examine the interaction between training aspect and transfer effect; however, in the present study, we did not conduct this analysis because the number of studies is not enough. Finally, in accordance with the last point, we did not conduct multivariate analysis in the present study because of lack of effect sizes; therefore, we could not reach a conclusion on the interaction between different moderators. Future empirical works and meta-analysis are needed to investigate the interaction between different moderators.

## Conclusions

Collectively, our findings contribute to the literature related to computer-based training on EFs in several key ways. The present results provide a meta-analysis confirmation of the different training effects of computer-based EF training. First, computer-based training can increase EFs in children, especially of working memory and inhibition, but not flexibility. Second, computer-based training benefits both near-transfer and far-transfer effects, whereas near-transfer effect was marginally significantly higher than far-transfer effect. Third, the training effects of the standard training approach were more robust than game-based training. In addition, typically developing children benefit more from computer-based training. Finally, the mean sample age impacted the training effects. The current findings demonstrate that computer-based training can serve as an effective training tool, but the benefits and transfer effects vary based on the different population groups.

## Author Contributions

YC conceived this study and was involved in conducting the meta-analysis, processing data, and writing the manuscript. TH and JH was involved in conceiving this study, data coding, and writing the manuscript. XX participated in data analysis and writing the manuscript. YW participated in data interpretation and writing the manuscript. All authors contributed to the article and approved the submitted version.

## Conflict of Interest

The authors declare that the research was conducted in the absence of any commercial or financial relationships that could be construed as a potential conflict of interest.
