# Supplementary material for: Effects and Moderators of Computer-Based Training on Children's Executive Functions: A Systematic Review and Meta-Analysis
Source: Front Psychol. 2020 Nov 26;11:580329. doi: 10.3389/fpsyg.2020.580329 (PMC7726355; doi:10.3389/fpsyg.2020.580329)
Supplement: Supplementary file 1 [file Table_1.doc]

**Table 1 Summary of the studies included into the present meta-analysis**

| **First Author** | **Publication year** | **Mean age(year)** | **Clinical risk status** | **Intervention condition** | **Control condition** | **Number of session** | **Training time(minute)** | **Outcome measure** | **Transfer effect** | **Posttest(Hedge's g)** | **Follow-up test(Hedge's g)** |
| --- | --- | --- | --- | --- | --- | --- | --- | --- | --- | --- | --- |
| Ackermann（2018） | 2018 | 13.8 | Atypical: diagnosed with ADHD | Game-based: WM training using “Cogmed ”(n = 18) | Passive control (n = 10) | 20 |  | working memory(3 measures): |  |  |  |
|  |  |  |  |  |  |  |  | - Digit span forward | Near transfer | yes(0.62) |  |
|  |  |  |  |  |  |  |  | - Digit span backward | Near transfer | yes(0.77) |  |
|  |  |  |  |  |  |  |  | - Spatial span forward | Near transfer | yes(0.94) |  |
|  |  |  |  |  |  |  |  | Inhibition(3 measures)： |  |  |  |
|  |  |  |  |  |  |  |  | - Stroop task | Far transfer | yes(0.05) |  |
|  |  |  |  |  |  |  |  | - Go/no-go task-Errors | Far transfer | yes(-0.12) |  |
| Alloway | 2013 | 10.76 | Atypical: diagnosed with learning difficulties | Game-based: WM training using “Jungle Memory” (n = 23) | Active control (n = 32) | 32 |  | Working memory (2 measures): |  |  |  |
| Contrast 1 |  |  |  |  |  |  |  | - Mix of backward digit recall and processing letter recall | Near transfer | Yes(0.85) | Yes(1.35) |
|  |  |  |  |  |  |  |  | - Shape recall task | Near transfer | Yes(0.42) | Yes(1.39) |
| Alloway | 2013 | 10.76 | Atypical: diagnosed with learning difficulties | Game-based: WM training using “Jungle Memory” (n = 23) | Passive control (n = 39) | 32 |  | Working memory (2 measures): |  |  |  |
| Contrast 2 |  |  |  |  |  |  |  | - Mix of backward digit recall and processing letter recall | Near transfer | Yes(0.59) | Yes(0.76) |
|  |  |  |  |  |  |  |  | - Shape recall task | Near transfer | Yes(0.40) | Yes(1.59) |
| Bikic | 2018 | 9.955 | Atypical: diagnosed with ADHD | Standard: CF training (n = 35) | Active control (n = 35) | 20 |  | Woriking memory(1 measure): |  |  |  |
|  |  |  |  |  |  |  |  | - Spatial working memory- between errors | Near transfer | yes(0.32) | Yes(0.10) |
|  |  |  |  |  |  |  |  | Flexibility(2 measures): |  |  |  |
|  |  |  |  |  |  |  |  | - AST-total omission errors | Near transfer | yes(0.08) | Yes(0.21) |
|  |  |  |  |  |  |  |  | - IED-errors | Near transfer | yes(0.45) | Yes(0.12) |
|  |  |  |  |  |  |  |  | Inhibition（1 measure）： |  |  |  |
|  |  |  |  |  |  |  |  | - SST-errors | Near transfer | yes(0.42) | Yes(0.33) |
| Bigorra | 2015 | 8.79 | Atypical: diagnosed with ADHD | Game-based: WM training using “Cogmed” (n = 31) | Active control (n = 30) | 25 | 1000 | Working memory (2 measures): |  |  |  |
|  |  |  |  |  |  |  |  | - Letter number sequencing | Near transfer | Yes(0.57) | Yes(0.08) |
|  |  |  |  |  |  |  |  | - Spatial recall | Near transfer | Yes(0.711) | Yes(0.17) |
|  |  |  |  |  |  |  |  | Flexibility(2 measures): |  |  |  |
|  |  |  |  |  |  |  |  | - Tower of London | Far transfer | Yes(0.016) | Yes(-0.18) |
|  |  |  |  |  |  |  |  | - WCST-64-perseverative | Far transfer | Yes(-0.15) | Yes(-0.20) |
|  |  |  |  |  |  |  |  | Inhibition(1 measure): |  |  |  |
|  |  |  |  |  |  |  |  | - CPT-commission errors | Far transfer | Yes(0.29) | Yes(0.08) |
|  |  |  |  |  |  |  |  | Flexibility(1 measure): |  |  |  |
|  |  |  |  |  |  |  |  | - Trial Making Test | Far transfer | Yes(-0.46) | Yes(-0.01) |
| Blakey | 2015 | 4 | Typical | Standard: WM and IC training (n = 26) | Active control (n = 28) | 4 | 80 | Flexibility(2 measures): | Far transfer |  |  |
|  |  |  |  |  |  |  |  | - FIST | Far transfer | Yes(0.096) | Yes(0.05) |
|  |  |  |  |  |  |  |  | - SwIFT mixrd switch | Far transfer | Yes(0.13) | Yes(0.15) |
|  |  |  |  |  |  |  |  | Working memory (1 measure): |  |  |  |
|  |  |  |  |  |  |  |  | - Backward word | Near transfer | Yes(0.52) | Yes(0.87) |
|  |  |  |  |  |  |  |  | Inhibition(1 measure): |  |  |  |
|  |  |  |  |  |  |  |  | - Peg tapping | Near transfer | Yes(0.0075) | Yes(0.39) |
| de Vries | 2015 | 10 | Typical | Game-based: CF training (n = 36) | Active control (n = 29) | 25 |  | Flexibility(2 measures): |  |  |  |
| Contrast 1 |  |  |  |  |  |  |  | - Geder-emotion switch | Near transfer | Yes(0.094) | Yes(0.06) |
|  |  |  |  |  |  |  |  | - Number-gnome switch | Near transfer | Yes(-0.099) | Yes(-0.35) |
|  |  |  |  |  |  |  |  | Working memory (1 measure): |  |  |  |
|  |  |  |  |  |  |  |  | - N-back | Far transfer | Yes(0.057) | Yes(0.21) |
|  |  |  |  |  |  |  |  | Inhibition(1 measure): |  |  |  |
|  |  |  |  |  |  |  |  | - Stop-task(comission errors) | Far transfer | Yes(0.092) | Yes(0.33) |
| de Vries | 2015 | 10 | Typical | Game-based: WM training (n= 40) | Active control (n = 29) | 25 |  | Flexibility(2 measures): |  |  |  |
| Contrast 2 |  |  |  |  |  |  |  | - Geder-emotion switch | Far transfer | Yes(-0.178) | Yes(-0.07) |
|  |  |  |  |  |  |  |  | - Number-gnome switch | Far transfer | Yes(-0.0916) | Yes(0) |
|  |  |  |  |  |  |  |  | Working memory (1 measure): |  |  |  |
|  |  |  |  |  |  |  |  | - N-back | Near transfer | Yes(0.035) | Yes(0.01) |
|  |  |  |  |  |  |  |  | Inhibition(1 measure): |  |  |  |
|  |  |  |  |  |  |  |  | - Stop-task(comission errors) | Far transfer | Yes(-0.127) | Yes(-0.18) |
| Dörrenbächer | 2014 | 9.644 | Typical | Standard: EF training using “big thing and small thing task ”(n = 13) | Active control (n = 14) |  |  | Working memory (2 measures): |  |  |  |
| contrast 1 |  |  |  |  |  |  |  | - Backward digit recall |  | Yes(0.05) |  |
|  |  |  |  |  |  |  |  | - Counting span |  | Yes(0.16) |  |
|  |  |  |  |  |  |  |  | Inhibition (3 measures): |  |  |  |
|  |  |  |  |  |  |  |  | - AX-CPT |  | Yes(0.37) |  |
|  |  |  |  |  |  |  |  | - BX-CPT |  | Yes(0.21) |  |
|  |  |  |  |  |  |  |  | - Color stroop |  | Yes(0.14) |  |
|  |  |  |  |  |  |  |  | Flexibility (1 measure): |  |  |  |
|  |  |  |  |  |  |  |  | - Switching |  | Yes(0.29) |  |
| Dörrenbächer | 2014 | 9.644 | Typical | Standard: EF training“big thing and small thing task ”(n = 14) | Active control (n = 13) |  |  | Working memory (2 measures): |  |  |  |
| contrast 2 |  |  |  |  |  |  |  | - Backward digit recall |  | Yes(0.41) |  |
|  |  |  |  |  |  |  |  | - Counting span |  | Yes(0.34) |  |
|  |  |  |  |  |  |  |  | Inhibition (3 measures): |  |  |  |
|  |  |  |  |  |  |  |  | - AX-CPT |  | Yes(0.86) |  |
|  |  |  |  |  |  |  |  | - BX-CPT |  | Yes(-0.39) |  |
|  |  |  |  |  |  |  |  | - Color stroop |  | Yes(-0.82) |  |
|  |  |  |  |  |  |  |  | Flexibility (1 measure): |  |  |  |
|  |  |  |  |  |  |  |  | - Switching |  | Yes(-0.04) |  |
| Dovis | 2015 | 10.6 | Atypical: diagnosed with ADHD | Game-based: EF training using “Briangame Brian ”(n = 31) | Active control (n = 30) | 25 | 750 | Working memory(2 measures): |  |  |  |
| Contrast 1 |  |  |  |  |  |  |  | - Digit Span | Near transfer | Yes(0) | Yes(0.13) |
|  |  |  |  |  |  |  |  | - CBTT-forward | Near transfer | Yes(0.748) | Yes(0.61) |
|  |  |  |  |  |  |  |  | - CBTT-backward | Near transfer | Yes(0.34) | Yes(0.26) |
|  |  |  |  |  |  |  |  | Inhibition(2 measures): |  |  |  |
|  |  |  |  |  |  |  |  | - Stop task | Near transfer | Yes(0.71) | Yes(0.73) |
|  |  |  |  |  |  |  |  | - Stroop | Near transfer | Yes(0.11) | Yes(0.6) |
|  |  |  |  |  |  |  |  | Flexibility(1 measure): |  |  |  |
|  |  |  |  |  |  |  |  | - TMT | Near transfer | Yes(-0.90) | Yes(0.04) |
| Dovis | 2015 | 10.6 | Atypical: diagnosed with ADHD | Game-based : IC and CF training using “Briangame Brian” (n = 28) | Active control (n = 30) | 25 | 750 | Working memory(3 measures): |  |  |  |
| Contrast 2 |  |  |  |  |  |  |  | - Digit Span | Far transfer | Yes(0) | Yes(0.03) |
|  |  |  |  |  |  |  |  | - CBTT-forward | Far transfer | Yes(0) | Yes(0.04) |
|  |  |  |  |  |  |  |  | - CBTT-backward | Far transfer | Yes(0.091) | Yes(0.04) |
|  |  |  |  |  |  |  |  | Inhibition(2 measures): |  |  |  |
|  |  |  |  |  |  |  |  | - Stop task | Near transfer | Yes(0.71) | Yes(-0.64) |
|  |  |  |  |  |  |  |  | - Stroop | Near transfer | Yes(0.26) | Yes(-0.31) |
|  |  |  |  |  |  |  |  | Flexibility(1 measure): |  |  |  |
|  |  |  |  |  |  |  |  | - TMT | Near transfer | Yes(0.099) | Yes(0.13) |
| Dunning | 2013 | 8.417 | Atypical: diagnosed with low WM | Game-based: WM training using “Cogmed” (n = 34) | Active control (n = 30) | 25 | 750 | Working memory(2 measures): |  |  |  |
| Contrast 1 |  |  |  |  |  |  |  | - Backward digit recall,Listening recall | Near transfer | Yes(0.56) |  |
|  |  |  |  |  |  |  |  | - Mr.X,odd one out | Near transfer | Yes(0.46) |  |
|  |  |  |  |  |  |  |  | Inhibition(1 measure): |  |  |  |
|  |  |  |  |  |  |  |  | - CPT-commission error | Far transfer | Yes(0.403) |  |
| Dunning | 2013 | 8.417 | Atypical: diagnosed with low WM | Game-based: WM training using “Cogmed” (n = 34) | Passive control (n = 30) | 25 | 750 | Working memory(2 measures): |  |  |  |
| Contrast 2 |  |  |  |  |  |  |  | - Backward digit recall,Listening recall | Near transfer | Yes(0.00169) |  |
|  |  |  |  |  |  |  |  | - Mr.X,odd one out | Near transfer | Yes(0.97) |  |
|  |  |  |  |  |  |  |  | Inhibition(1 measure): |  |  |  |
|  |  |  |  |  |  |  |  | - CPT-commission error | Far transfer | Yes(0.30) |  |
| Egeland | 2013 | 10.4 | Atypical: diagnosed with ADHD | Standard: WM training using “Cogmed” (n = 33) | Active control (n = 34) | 25 | 750 | Inhibition(2 measures)： |  |  |  |
|  |  |  |  |  |  |  |  | - Color Word - controlled attention (Stroop test) | Far transfer | yes(0.05) | Yes(0.1) |
|  |  |  |  |  |  |  |  | - CPT - hyperactivity/impulsivity | Far transfer | yes(0.36) | Yes(0.28) |
|  |  |  |  |  |  |  |  | Flexibility(1 measure): |  |  |  |
|  |  |  |  |  |  |  |  | - TMT | Far transfer | yes(0.12) | Yes(0.04) |
| Espinet | 2012 | 3.417 | Typical | Standard : CF training (n = 15) | Active control (n = 14) | 2 | 30 | Flexibility (1 measure): |  |  |  |
| Contrast 1 |  |  |  |  |  |  |  | - Dimensional Change Card Sort (DCCS) |  | Yes(0.84) |  |
| Espinet | 2012 | 3.417 | Typical | Standard : CF training (n = 14) | Active control (n = 14) | 2 | 30 | Flexibility (1 measure): |  |  |  |
| Contrast 2 |  |  |  |  |  |  |  | - Dimensional Change Card Sort (DCCS) |  | Yes(0.86) |  |
| Espinet | 2012 | 3.417 | Typical | Standard : CF training (n = 20) | Active control (n = 16) | 2 | 30 | Flexibility (1 measure): |  |  |  |
| Contrast 3 |  |  |  |  |  |  |  | - Dimensional Change Card Sort (DCCS) |  | Yes(1.00) |  |
| Espinet | 2012 | 3.417 | Typical | Standard : CF training (n = 20) | Active control (n = 20) | 2 | 30 | Flexibility (1 measure): |  |  |  |
| Contrast 4 |  |  |  |  |  |  |  | - Dimensional Change Card Sort (DCCS) |  | Yes(1.15) |  |
| Gary | 2012 | 14.32 | Atypical: diagnosed with ADHD | Standard: WM training (n = 32) | Active control (n = 20) | 20 | 900 | Working memory(2 measures): |  |  |  |
|  |  |  |  |  |  |  |  | - Digit Span Forward | Near transfer | yes(0.23) |  |
|  |  |  |  |  |  |  |  | - Cambridge Neuropsychological Testing Automated Battery | Near transfer | yes(0.52) |  |
| Goldin | 2014 | 6.5 | Atypical: diagnosed with low-SES | Game-based: WM and IC training (n = 73) | Active control (n = 38) | 23 | 345 | Inhibition (2 measures): |  |  |  |
|  |  |  |  |  |  |  |  | - The Attention Network Test for children | Near transfer | Yes(0.09) |  |
|  |  |  |  |  |  |  |  | - The Heart-Flower Stroop task (fix incongruent) | Near transfer | Yes(0.02) |  |
|  |  |  |  |  |  |  |  | Flexibility  (3 measures): |  |  |  |
|  |  |  |  |  |  |  |  | - Tower of London | Far transfer | Yes(0.11) |  |
|  |  |  |  |  |  |  |  | - The Heart-Flower Stroop task (mix congruent) | Far transfer | Yes(0.17) |  |
|  |  |  |  |  |  |  |  | - The Heart-Flower Stroop task (mix incongruent) | Far transfer | Yes(0.37) |  |
| Hessl | 2019 | 13.76 | Atypical: diagnosed with fragile X syndrome | Game-based: WM training using “Cogmed” (n = 49) | Active control (n = 48) | 25 | 945 | woriking memory(2 measure): |  |  |  |
|  |  |  |  |  |  |  |  | - Digit Span | Near transfer | yes(0.002) |  |
|  |  |  |  |  |  |  |  | - Visual Working Memory Composite | Near transfer | yes(-0.11) |  |
|  |  |  |  |  |  |  |  | Flexibility(1 measure): |  |  |  |
|  |  |  |  |  |  |  |  | - Flexibility False Alarms | Far transfer | yes(0.95) |  |
|  |  |  |  |  |  |  |  | Inhibition(1 measure): |  |  |  |
|  |  |  |  |  |  |  |  | - Go/No-go - false alarms | Far transfer | yes(0.76) |  |
| Holmes | 2009 | 10 | Typical | Standard: WM training (n = 22) | Passive control (n = 20) | 20 | 700 | Working memory (2 measures): |  |  |  |
|  |  |  |  |  |  |  |  | - Verbal WM |  | Yes(0.72) | Yes(0.1) |
|  |  |  |  |  |  |  |  | - Visuo-spatial WM |  | Yes(0.43) | Yes(0.29) |
| Hovik | 2013 | 10.5 | Atypical: diagnosed with ADHD | Standard: WM training using “Cogmed” (n = 33) | Passive control (n = 34) | 25 | 750 | Working memory (3 measures): |  |  |  |
|  |  |  |  |  |  |  |  | - Auditory WM: (Digit span forward + Digit Span backward) | Near transfer | Yes(0) |  |
|  |  |  |  |  |  |  |  | - Visual WM: (Leiter R forward + Leiter R backward) | Near transfer | Yes(0.35) |  |
|  |  |  |  |  |  |  |  | - Manipulation WM (Letter-Number + Sentence Span) | Near transfer | Yes(0.13) |  |
| Klingberg | 2002 | 11 | Atypical: diagnosed with ADHD | Standard: WM training task (n = 7) | Active control (n = 7) | 25 | 607.5 | Working memory (2 measures): |  |  |  |
|  |  |  |  |  |  |  |  | - Trained version of the visuo-spatial WM task | Near transfer | Yes(4.86) |  |
|  |  |  |  |  |  |  |  | - Span board | Near transfer | Yes(4.34) |  |
|  |  |  |  |  |  |  |  | Inhibition (1 measure): |  |  |  |
|  |  |  |  |  |  |  |  | - Stroop task | Far transfer | Yes(2.18) |  |
| Klingberg | 2005 | 9.8 | Atypical: diagnosed with ADHD | Game-based: WM trlaining using “Cogmed” (n = 20) | Active control (n = 24) | 25 | 1000 | Working memory (2 measures): |  |  |  |
|  |  |  |  |  |  |  |  | - The span-board task from the WAIS-RNI testing battery | Near transfer | Yes(1.03) | Yes(1) |
|  |  |  |  |  |  |  |  | - Digit-span from the WISC-III testing battery | Near transfer | Yes(0.43) | Yes(0.44) |
|  |  |  |  |  |  |  |  | Inhibition (1 measure): |  |  |  |
|  |  |  |  |  |  |  |  | - Stroop task | Far transfer | Yes(0.13) | Yes(0.2) |
| Kuhn | 2014 | 9 | Atypical: diagnosed with ADHD | Standard: WM training task (n = 19) | Passive control (n = 20) | 15 | 300 | Working memory(2 measures): |  |  |  |
|  |  |  |  |  |  |  |  | - DEMAT | Near transfer | yes(0.32) |  |
|  |  |  |  |  |  |  |  | - Spatial WM | Near transfer | yes(-0.27) |  |
| Liu | 2016 | 4.87 | Typical | Game-based: IC training using "Fruit Ninja" (n = 16) | Active control (n = 20) | 12 | 180 | Working memory (2 measures): |  |  |  |
|  |  |  |  |  |  |  |  | - The digit span forward subtests of the WPPSI-III | Far transfer | Yes(0.34) |  |
|  |  |  |  |  |  |  |  | - The digit span backward subtests of the WPPSI-III | Far transfer | Yes(0.62) |  |
|  |  |  |  |  |  |  |  | Inhibition (2 measures): |  |  |  |
|  |  |  |  |  |  |  |  | - The adapted version of the day-night Stroop Task | Near transfer | Yes(0.60) |  |
|  |  |  |  |  |  |  |  | - The go/no-go task | Near transfer | Yes(-0.19) |  |
| Luo | 2013 | 10.6 | Atypical: diagnosed with developmental dyslexia | Standard: WM training task (n = 15) | Active control (n = 15) | 25 | 1000 | Working memory (2 measures): |  |  |  |
|  |  |  |  |  |  |  |  | - Digit span task | Near transfer | Yes(0.53) |  |
|  |  |  |  |  |  |  |  | - Word span task | Near transfer | Yes(0.68) |  |
|  |  |  |  |  |  |  |  | Inhibition (1 measure'): |  |  |  |
|  |  |  |  |  |  |  |  | - Stroop task | Near transfer | Yes(0.00) |  |
| Nutley | 2011 | 4 | Typical | Game-based: WM training using “Cogmed” (n = 24) | Active control (n = 25) | 25 | 375 | Working memory (3 measures): |  |  |  |
|  |  |  |  |  |  |  |  | - Visuo-spatial grid task | Near transfer | Yes(1.56) |  |
|  |  |  |  |  |  |  |  | - The Odd One Out from the AWMA | Near transfer | Yes(0.55) |  |
|  |  |  |  |  |  |  |  | - The Word Span | Near transfer | Yes(0.50) |  |
| Paul | 2018 | 7.84 | Typical | Standard: IC training task (n = 33) | Active control (n = 33) | 10 | 450 | Working memory (1 measure): |  |  |  |
| Contrast 1 |  |  |  |  |  |  |  | - Working Memory Span backwards subtests of the WISC | Far transfer | Yes(0.49) |  |
|  |  |  |  |  |  |  |  | Inhibition (2 measures): |  |  |  |
|  |  |  |  |  |  |  |  | - Simon Says task | Near transfer | Yes(0.30) |  |
|  |  |  |  |  |  |  |  | - Child-friendly version of the flanker task | Near transfer | Yes(0.05) |  |
| Paul | 2018 | 6.56 | Typical | Standard: IC training task (n = 31) | Active control (n = 33) | 10 | 450 | Working memory (1 measure): |  |  |  |
| Contrast 2 |  |  |  |  |  |  |  | - Working Memory Span backwards subtests of the WISC | Far transfer | Yes(0.15) |  |
|  |  |  |  |  |  |  |  | Inhibition (2 measures): |  |  |  |
|  |  |  |  |  |  |  |  | - Simon Says task | Near transfer | Yes(0.26) |  |
|  |  |  |  |  |  |  |  | - Child-friendly version of the flanker task | Near transfer | Yes(0.27) |  |
|  |  |  |  |  |  |  |  | Flexibility (1 measure): |  |  |  |
|  |  |  |  |  |  |  |  | - Trail Making tests (TMT) | Far transfer | Yes(0.12) |  |
| Prins | 2011 | 9.47 | Atypical: diagnosed with ADHD | Game-based: WM training (n = 27) | Active control (n = 24) | 3 | 90 | Working memory: |  |  |  |
|  |  |  |  |  |  |  |  | - Corsi Block-Tapping Test | Near transfer | yes(0.637) |  |
| Rojas | 2015 | 4.36 | Typical | Standard: WM training task (n = 144) | Passive control  (n = 124) | 16 | 480 | Working memory(2 measures): |  |  |  |
|  |  |  |  |  |  |  |  | - Phonological WM tasks | Near transfer | yes(0.933) |  |
|  |  |  |  |  |  |  |  | - Visuospatial WM tasks | Near transfer | yes(0.933) |  |
| Rueda | 2012 | 5.39 | Typical | Standard: IC training task (n = 19) | Active control (n = 18) | 10 | 450 | Inhibition (4 measures): |  |  |  |
|  |  |  |  |  |  |  |  | - Child ANT | Near transfer | Yes(0.20) | Yes(0.69) |
|  |  |  |  |  |  |  |  | - Delay of gratification(self) | Near transfer | Yes(0.61) | Yes(0.43) |
|  |  |  |  |  |  |  |  | - Delay of gratification(other) | Near transfer | Yes(0.42) | Yes(0.23) |
|  |  |  |  |  |  |  |  | - Iowa Gambling Task | Near transfer | Yes(0.35) | Yes(0.32) |
| Sánchez-Pérez | 2018 | 9.08 | Typical | Standard: WM training task (n = 51) | Active control (n = 53) | 13 | 390 | working memory(2 measures): |  |  |  |
|  |  |  |  |  |  |  |  | - Digit span forward | Near transfer | yes(0.24) |  |
|  |  |  |  |  |  |  |  | - Digit span backward | Near transfer | yes(0.01) |  |
|  |  |  |  |  |  |  |  | Inhibition(1 measure)： |  |  |  |
|  |  |  |  |  |  |  |  | - Go/no-go task-Errors | Far transfer | yes(0.90) |  |
|  |  |  |  |  |  |  |  | Flexibility(1 measure): |  |  |  |
|  |  |  |  |  |  |  |  | - Shifting (dots task) | Far transfer | yes(-0.24) |  |
| Sánchez-Pérez | 2019 | 9.06 | Typical | Standard: WM training task (n = 33) | Active control (n = 23) | 13 | 390 | Inhibition(1 measure)： |  |  |  |
|  |  |  |  |  |  |  |  | - Go/no-go task-Errors | Far transfer | yes(0.97) |  |
| St Clair | 2010 | 6.88 | Typical | Game-based: WM training using "Memory Booster" (n = 117) | Passive control  (n = 137) | 2 | 60 | Working memory(3 measures): |  |  |  |
|  |  |  |  |  |  |  |  | - Digit recall task | Near transfer | yes(0.38) |  |
|  |  |  |  |  |  |  |  | - Listening recall task | Near transfer | yes(1.146) |  |
|  |  |  |  |  |  |  |  | - Block recall task | Near transfer | yes(0.178) |  |
| Thorell | 2009 | 4.7 | Typical | Game-based: WM and IC training using “Cogmed” (n = 18) | Active control (n = 14) | 25 | 375 | Working memory (2 measures): |  |  |  |
| Contrast 1 |  |  |  |  |  |  |  | - The Span board task from WAIS-R-NI | Near transfer | Yes(-1.45) |  |
|  |  |  |  |  |  |  |  | - A word span task | Near transfer | Yes(-0.51) |  |
|  |  |  |  |  |  |  |  | Inhibition (2 measures): |  |  |  |
|  |  |  |  |  |  |  |  | - Day-Night Stroop Task | Near transfer | Yes(0.16) |  |
|  |  |  |  |  |  |  |  | - Go/no-go task | Near transfer | Yes(0.12) |  |
| Thorell | 2009 | 4.7 | Typical | Game-based: WM and IC training using “Cogmed” (n = 18) | Passive control (n = 15) | 25 | 375 | Working memory (2 measures): |  |  |  |
| Contrast 2 |  |  |  |  |  |  |  | - The Span board task from WAIS-R-NI | Near transfer | Yes(-1.01) |  |
|  |  |  |  |  |  |  |  | - A word span task | Near transfer | Yes(-0.52) |  |
|  |  |  |  |  |  |  |  | Inhibition (2 measures): |  |  |  |
|  |  |  |  |  |  |  |  | - Day-Night Stroop Task | Near transfer | Yes(0.06) |  |
|  |  |  |  |  |  |  |  | - Go/no-go task | Near transfer | Yes(-0.27) |  |
| Thorell | 2009 | 4.7 | Typical | Game-based: WM and IC training using “Cogmed” (n = 17) | Active control (n = 14) | 25 | 375 | Working memory (2 measures): |  |  |  |
| Contrast 3 |  |  |  |  |  |  |  | - The Span board task from WAIS-R-NI | Near transfer | Yes(0.20) |  |
|  |  |  |  |  |  |  |  | - A word span task | Near transfer | Yes(0.31) |  |
|  |  |  |  |  |  |  |  | Inhibition (2 measures): |  |  |  |
|  |  |  |  |  |  |  |  | - Day-Night Stroop Task | Near transfer | Yes(0.42) |  |
|  |  |  |  |  |  |  |  | - Go/no-go task | Near transfer | Yes(-0.09) |  |
| Thorell | 2009 | 4.7 | Typical | Game-based: WM and IC training using “Cogmed” (n = 17) | Passive control (n = 16) | 25 | 375 | Working memory (2 measures): |  |  |  |
| Contrast 4 |  |  |  |  |  |  |  | - The Span board task | Near transfer | Yes(0.30) |  |
|  |  |  |  |  |  |  |  | - A word span task | Near transfer | Yes(0.30) |  |
|  |  |  |  |  |  |  |  | Inhibition (2 measures): |  |  |  |
|  |  |  |  |  |  |  |  | - Day-Night Stroop Task | Near transfer | Yes(0.33) |  |
|  |  |  |  |  |  |  |  | - Go/no-go task (Berlin & Bohlin, 2002) | Near transfer | Yes(-0.25) |  |
| Verbeken | 2012 | 11.46 | Typical | Game-based: WM and IC training using "Braingame Brain" (n = 21) | passive control (n = 22) | 25 | 1000 | Working memory(2 measures): |  |  |  |
|  |  |  |  |  |  |  |  | - Corsi Blocking Tapping Test | Far transfer | yes(0.713) |  |
|  |  |  |  |  |  |  |  | - Corsi Blocking Tapping Test-BW | Far transfer | yes(0.503) |  |
| Wong | 2014 | 8.11 | Atypical:diagnosed with poor working memory | Standard: WM and IC training task (n = 26) | Passive control (n = 25) | 23 | 805 | Working memory (2 measures): |  |  |  |
|  |  |  |  |  |  |  |  | - The span-board task from the WAIS-RNI testing battery | Near transfer | Yes(0.56) | Yes(-0.23) |
|  |  |  |  |  |  |  |  | - Digit Span | Far transfer | Yes(-0.21) | Yes(-0.51) |
|  |  |  |  |  |  |  |  | Inhibition (1 measure): |  |  |  |
|  |  |  |  |  |  |  |  | - The Stroop interference task | Near transfer | Yes(-0.30) | Yes(0.58) |
| Zhang | 2018 | 6.13 | Typical | Standard: WM training (n = 20) | Active control (n = 25) | 20 | 300 | Working memory (2 measure): |  |  |  |
| Contrast 1 |  |  |  |  |  |  |  | - Backward digit recall | Near transfer | Yes(0.125) | Yes(0.11) |
|  |  |  |  |  |  |  |  | - AX-CPT | Near transfer | Yes(0.248) | Yes(0.04) |
| Zhang | 2018 | 6.13 | Typical | Standard: WM training (n = 20) | Active control (n = 22) | 20 | 300 | Working memory (2 measure): |  |  |  |
| Contrast 2 |  |  |  |  |  |  |  | - Backward digit recall | Near transfer | Yes(0.0063) | Yes(1.2) |
|  |  |  |  |  |  |  |  | - AX-CPT | Near transfer | Yes(0.79) | Yes(0.22) |
| Zhang | 2018 | 6.13 | Typical | Standard: IC training (n = 21) | Active control (n = 25) | 20 | 300 | Inhibition(2 measure): |  |  |  |
| Contrast 3 |  |  |  |  |  |  |  | - Backward digit recall | Near transfer | Yes(0.28) | Yes(-0.35) |
|  |  |  |  |  |  |  |  | - AX-CPT | Near transfer | Yes(-0.64) | Yes(1.21) |
| Zhang | 2018 | 6.13 | Typical | Standard: IC training (n = 21) | Active control (n = 22) | 20 | 300 | Inhibition(2 measure): |  |  |  |
| Contrast 4 |  |  |  |  |  |  |  | - Backward digit recall | Near transfer | Yes(0.103) | Yes(-0.65) |
| Weerdmeester | 2016 | 9.77 | Atypical: diagnosed with ADHD | Game-based: IC training using "Adventurous Dreaming Highflying Dragon" (n = 32) | Passive control (n = 34) | 6 | 90 | Inhibition(3 measures)： |  |  |  |
|  |  |  |  |  |  |  |  | - Go/no go task | Near transfer | Yes(0.124) |  |
|  |  |  |  |  |  |  |  | - MABC-2-NL-fine motor skill | Far transfer | Yes(0.113) |  |
|  |  |  |  |  |  |  |  | - MABC-2-NL-gross motor skill | Near transfer | Yes(0.272) |  |

Note. WM = working memory, IC = inhibitory control, CF = cognitive flexibility, EF training = training included all three aspects of executive functions, ADHD = attention deficit hyperactivity disorder, SES = socioecomonic status.
